# A Unique Case of Adenoid Cystic Carcinoma of the Left Main Bronchus

**DOI:** 10.1155/DTE.1.237

**Published:** 1995

**Authors:** Toshiaki Kobayashi, Chimori Konaka, Hiroyuki Shibanuma, Hiromi Serizawa, Yoshirou Ebihara, Harubumi Kato

**Affiliations:** 1 Departments of Surgery Tokyo Medical College 6-7-1, Nishishinjuku Shinjuku-ku Tokyo 160 Japan; 2 Departments of Pathology Tokyo Medical College 6-7-1, Nishishinjuku Shinjuku-ku Tokyo 160 Japan

## Abstract

A unique case of adenoid cystic carcinoma of the left main bronchus is reported.


*Diagnostic and Therapeutic Endoscopy*, 1995, Vol. 1, pp. 237–240
Reprints available directly from the publisher
Photocopying permitted by license only

© 1995 Harwood Academic Publishers GmbH
Printed in Singapore

### A Unique Case of Adenoid Cystic Carcinoma of the Left Main Bronchus

TOSHIAKI KOBAYASHI,[1] CHIMORI KONAKA,[1] HIROYUKI SHIBANUMA,[1] HIROMI SERIZAWA,[2] YOSHIROU EBIHARA,[2] and HARUBUMI KATO[1]

[1]*Departments of Surgery and* [2]*Pathology, Tokyo Medical College, 6-7-1, Nishishinjuku, Shinjuku-ku, Tokyo 160, Japan*

*(Received September 9, 1994; in final form December 9, 1994)*

A unique case of adenoid cystic carcinoma of the left main bronchus is reported.

**KEY WORDS:** adenoid cystic carcinoma, lung cancer, bronchoscopy, pulmonary adenoid cystic carcinoma, bronchoscope

### INTRODUCTION

Pulmonary adenoid cystic carcinoma is a rare malignancy. Most pulmonary adenoid cystic carcinomas arise from the trachea or main bronchi, and adenoid cystic carcinoma is the most common or second most common malignant tumor in the trachea (1–3). Adenoid cystic carcinoma has better prognosis than other lung cancers, regardless of therapy (1). It shows polypoid and/or subepithelial growths, and its surface is variegated (4–6). However, its true nature is unknown, because it is so rare. We report a unique adenoid cystic carcinoma case.

### CASE REPORT

A 57-year-old male was referred to our hospital with a diagnosis of adenocarcinoma in the left main bronchus. He had been treated for asthma because of cough for 18 months at another hospital, and he consulted a second hospital with complaints of hemoptysis, dyspnea, and chest pain. Bronchoscopy revealed the disease to be adenocarcinoma in the left main bronchus. He had a smoking history of 20 cigarettes per day for more than 40 years. A chest x-ray film revealed complete atelectasis of the lung. Bronchoscopy showed the polypoid tumor completely occluding the left main bronchus.

Figure 1 shows the bronchoscopic findings on his first visit to our hospital. The long polypoid and uneven tumor protruded from the left main bronchus. The surface of the tumor was coarse and necrotic with no prominent vessel. The bronchial mucosa was irregular with invasion up to the last cartilage ring of the trachea. The distal margin of the tumor was not visible, because the bronchial lumen was occluded completely. Biopsy of the tumor showed it to be adenocarcinoma.

Photodynamic therapy (PDT) was performed (7) three times with a total dose of 1,170 J using an argon dye laser. The tumor decreased in size and complete fenestration was accomplished. Figure 2 shows the findings of the left main bronchus 21 days after the last PDT session. The tumor occluding the left main bronchus disappeared, and the bronchus peripheral to the site of the tumor could be observed and indicated that the peripheral lung was functional. The mucosal surface of the bronchus still was irregular and variegated with necrosis and tumor vessels, and the possibility of residual tumor was considered. The cancer invasion still was observed from the last tracheal cartilage ring to the center of the left main bronchus.

Twenty-four days after the last PDT treatment, tracheobronchoplasty was performed. The length of the resected trachea and bronchus was 3.2 mm. Figure 3 shows

Address for correspondence: Toshiaki Kobayashi, MD, Department of Surgery, Tokyo Medical College, 6-7-1, Nishishinjuku, Shinjuku-ku, Tokyo 160, Japan

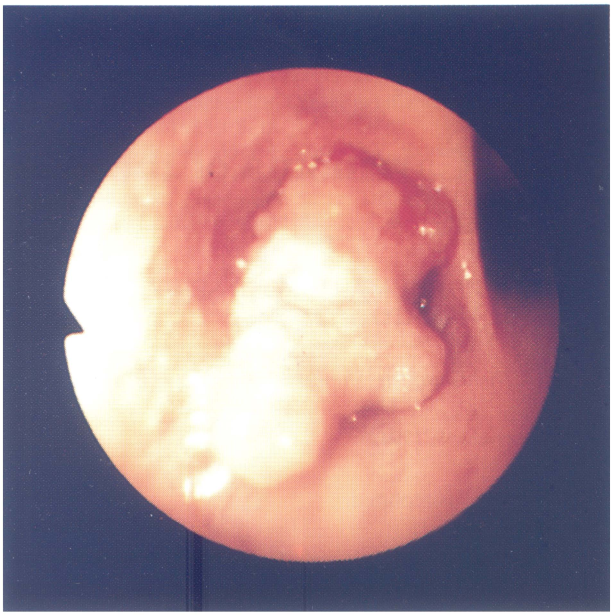

**Figure 1** Bronchoscopic findings of the tumor in the left main bronchus before PDT. The polypoid and uneven tumor are protruding from the left main bronchus.

the histological findings of the resected specimen. Most of the tumor showed a solid nest pattern and an anaplastic pattern, and it was difficult to distinguish the tumor from large cell lung cancer (Fig. 3a). A part of the tumor showed a cribriform pattern denoting adenoid cystic carcinoma (Figure 3b).

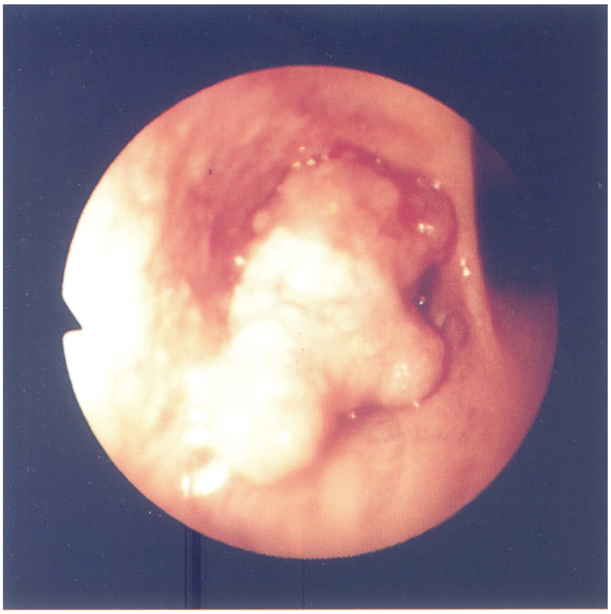

**Figure 2** Bronchoscopic findings of the left main bronchus 21 days after the last PDT.

Radiation therapy with a total dose of 60 Gy was given to the anastomosis site, because residual tumor cells were detected in the resected end of the trachea microscopically, which was not observed during surgery. Eleven months after surgery, metastasis was detected in the right adrenal gland. Subsequently, several metastases to bone and skin were detected. The skin metastasis was confirmed histologically. The patient died of massive hemoptysis 13 months after surgery. Repeated bronchoscopic brushing cytologies of the end-to-end anastomotic site did not show any evidence of recurrence of the tumor.

### DISCUSSION

Pulmonary adenoid cystic carcinoma is a rare malignancy among pulmonary neoplasms. As we can see from the fact that adenoid cystic carcinoma was initially considered to be a benign tumor, adenoid cystic carcinoma cases have a better prognosis than those of most lung cancer cases (1). The typical adenoid cystic carcinoma shows polypoid and subepithelial growths in the trachea or central bronchi (4,8), and the surface of the tumor is covered with the variegated mucosa (5,6). The best treatment of adenoid cystic carcinoma is surgical resection. The problem is the subepithelial invasion, which tends to cause residual tumor cells in the resected stump of the bronchi. Even with inoperable cases, relatively good survival can be expected by palliative treatment such as laser treatments. However, the true nature of adenoid cystic carcinoma is not sufficiently understood because of its rarity.

This case had a unique course. The short period to the detection of metastases was considered to be extremely early for an adenoid cystic carcinoma case. The microscopic findings also were unique. Cases with a solid nest pattern and anaplastic growth have a poorer prognosis than those with a cribriform pattern (9,10). A long survival was not expected in this case even without the massive hemoptysis, because most of the tumor was anaplastic and several metastases occurred rapidly.

The surface of the tumor was covered with necrosis, and almost no viable tumor was observed bronchoscopically. This also is a unique feature that could result in a misdiagnosis. Actually, the preoperative diagnosis of this case was poorly differentiated adenocarcinoma.

Residual tumor cells were detected at the resected margin of the trachea microscopically, although they were not observed during surgery. This is the problem of surgical resection of adenoid cystic carcinoma; however, we thought that local control had been attained by the radiation therapy because repeated bronchoscopic cytologies showed no evidence of the recurrence throughout the course.

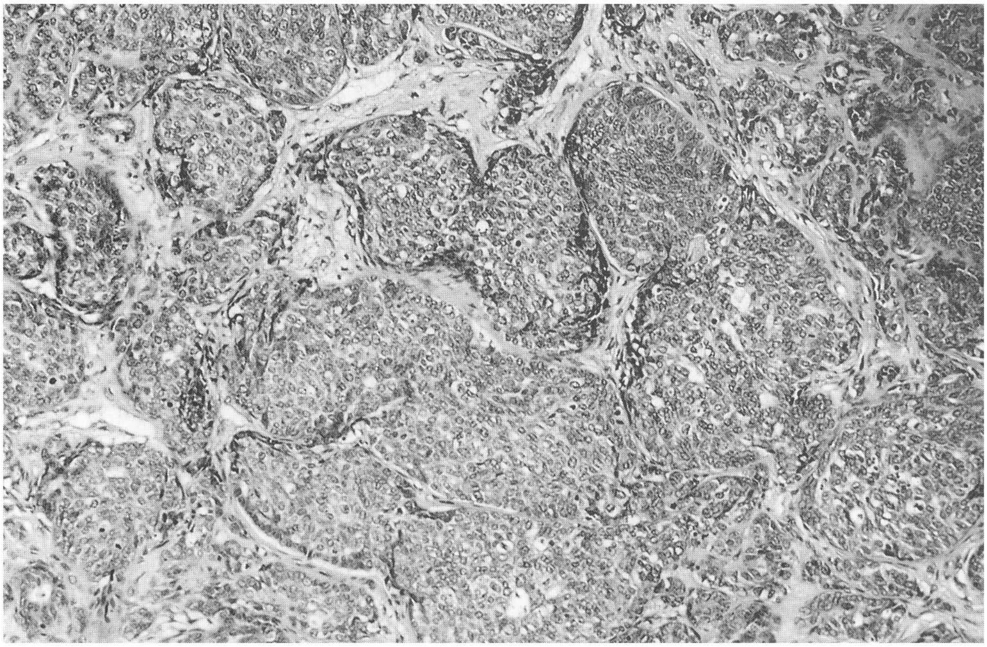

**Figure 3a**

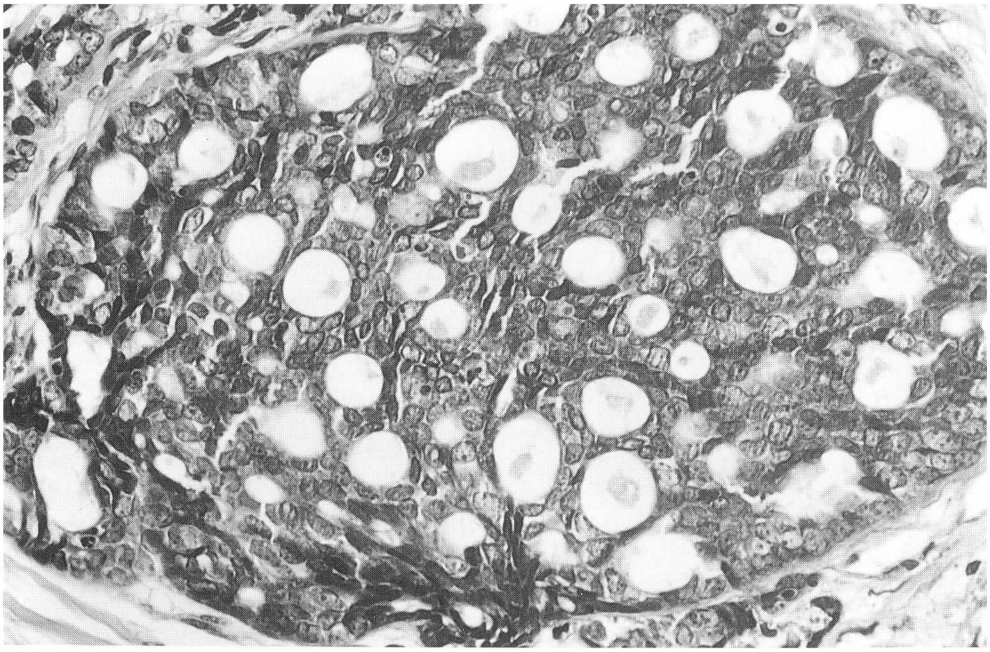

**Figure 3b** Histological findings of the tumor. Most of the tumor shows a solid nest pattern and an anaplastic pattern (Fig 3a), and part of the tumor shows a cribriform pattern (Fig 3b).

Although preoperative PDT could not eliminate all tumor cells from the bronchial wall, the initial purposes of PDT were attained, *i.e.*, the fenestration of the main bronchus and the confirmation of the distal end of the tumor to evaluate the length of the cancer invasion to decide on the indication of operation. In this respect, PDT is considered a good modality for adenoid cystic carcinoma in the airway.

This type of adenoid cystic carcinoma is problematic, because a polypoid tumor covered with necrosis in a central bronchus can be misdiagnosed as squamous cell carcinoma bronchoscopically. In addition, it is difficult to obtain a correct diagnosis when the biopsy specimen tends to consist of necrosis or anaplastic cells. With a diagnosis of other types of lung cancers, the subepithelial growth, which is characteristic of adenoid cystic carcinoma, tends to be underestimated, resulting in possible residual tumor cells at the end-to-end anastomosis site. These cases are considered essentially inoperable, and local control should be attempted by palliative treatment such as laser treatment and radiation therapy. Even if the complete resection of the tumor is fortuitously attained, future distant metastasis tends to be overlooked because of its histological diagnosis, namely, adenoid cystic carcinoma, which tends to recur locally. This results in the delay of systemic therapy. It is important to diagnose accurately adenoid cystic carcinoma and recognize that it can have an extremely poor outcome, as in this case.

### ACKNOWLEDGMENTS

The authors thank Professor J. Patrick Barron of the International Medical Communications Center, Tokyo Medical College, for his review of the manuscript.

### REFERENCES

1. Gelder CM, Hetzel MR. Primary tracheal tumors: a national survey. Thorax 1993;48:688–692.
2. Howard DJ, Haribhakti VV. Primary tumors of the trachea: analysis of clinical features and treatment results. Laryngol Otol 1994;108:230–232.
3. Manninen MP, Antila PJ, Pukander JS, Karma PH. Occurrence of tracheal carcinoma in Finland. Acta Otolaryngol (Stockholm) 1991;111:1162–1169.
4. Shimosato Y. Pulmonary neoplasms. In: Sternbergss, ed. Diagnostic surgical pathology. New York: Raven Press Ltd., 1989.
5. Kobayashi T, Konaka C, Kato H. Tracheobronchial tree. In: Kato H, ed. Electronic videoendoscopy. Chur, Switzerland: Harwood Academic Publishers, 1993.
6. Oho K, Amemiya R. Practical fiberoptic bronchoscopy. 2nd ed. Tokyo: Igaku-Shoin 1984.
7. Kato H, Konaka C, Ono J, et al. Preoperative laser photodynamic therapy in combination with operation in lung cancer. J Thorac Cardiovasc Surg 1985;90:420–429.
8. Kodama T, Shimosato Y, Kameya T. Histology and ultrastructure of bronchogenic and bronchial gland adenocarcinomas (including adenoid cystic and mucoepidermoid carcinomas) in relation to histogenesis. In: Shimosato Y, Melamed MR, Nettesheim P, eds. Morphogenesis of lung cancer. Boca Raton: CRC Press, 1982.
9. Nomori H, Kaseda S, Kobayashi K, et al. Adenoid cystic carcinoma of the trachea and main-stem bronchus, a clinical, histopathologic, and immunohistochemical study. J Thorac Cardiovasc Surg 1988;96:271–277.
10. Eby LS, Johnson DS, Baker HW. Adenoid cystic carcinoma of the head and neck. Cancer 1972;29:1160–1168.

